# Amphibious Transport of Fluids and Solids by Soft Magnetic Carpets

**DOI:** 10.1002/advs.202102510

**Published:** 2021-09-16

**Authors:** Ahmet F. Demirörs, Sümeyye Aykut, Sophia Ganzeboom, Yuki A. Meier, Robert Hardeman, Joost de Graaf, Arnold J. T. M. Mathijssen, Erik Poloni, Julia A. Carpenter, Caner Ünlü, Daniel Zenhäusern

**Affiliations:** ^1^ Complex Materials Department of Materials ETH Zurich Zurich 8093 Switzerland; ^2^ Institute for Theoretical Physics Center for Extreme Matter and Emergent Phenomena Utrecht University Princetonplein 5 Utrecht 3584 CC The Netherlands; ^3^ Department of Physics and Astronomy University of Pennsylvania Philadelphia PA 19104 USA; ^4^ Department of Chemistry Istanbul Technical University Istanbul 34469 Turkey; ^5^ Institut für Solartechnik SPF HSR University of Applied Sciences Rapperswil Rapperswil 8640 Switzerland

**Keywords:** artificial cilia, fluid dynamics, magnetic fields, self assembly, soft robots

## Abstract

One of the major challenges in modern robotics is controlling micromanipulation by active and adaptive materials. In the respiratory system, such actuation enables pathogen clearance by means of motile cilia. While various types of artificial cilia have been engineered recently, they often involve complex manufacturing protocols and focus on transporting liquids only. Here, soft magnetic carpets are created via an easy self‐assembly route based on the Rosensweig instability. These carpets can transport not only liquids but also solid objects that are larger and heavier than the artificial cilia, using a crowd‐surfing effect.This amphibious transportation is locally and reconfigurably tunable by simple micromagnets or advanced programmable magnetic fields with a high degree of spatial resolution. Two surprising cargo reversal effects are identified and modeled due to collective ciliary motion and nontrivial elastohydrodynamics. While the active carpets are generally applicable to integrated control systems for transport, mixing, and sorting, these effects can also be exploited for microfluidic viscosimetry and elastometry.

## Graphical abstract

Artificial magnetic cilia on a soft carpet can be made via a self‐assembly route based on the Rosensweig instability. Soft carpets can transport both liquids and solid objects that are larger and heavier than the artificial cilia, using a crowd‐surfing effect.This amphibious transportation is locally and reconfigurably tunable by simple micromagnets or advanced programmable magnetic fields with high spatial resolution.

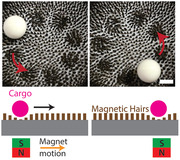

## 1 Introduction

Transport of solids and fluids is key to maintaining continuous processes in the microscopic and macroscopic world, e.g., mucociliary transport for the clearance of pathogens out of the respiratory system,^[^ ^1^ ^]^ the locomotion of reproductive cells by motile flagella,^[^ ^2^ ^]^ and the transport of goods in industry and everyday life.^[^ ^3^ ^]^ Transport generated by soft actuators is of interest in many areas ranging from soft robotics^[^ ^4^ ^]^ to drug delivery^[^ ^5^ ^]^ and microfluidics.^[^ ^6^ ^]^ In robotics, the transport of solid (and fragile) objects has been achieved using soft actuators.^[^ ^7^, ^8^, ^9^, ^10^ ^]^ Recent demonstrations thereof include pneumatic designs.^[^ ^11^, ^12^ ^]^ However, often these soft actuators have to be driven by complicated algorithms^[^ ^12^, ^13^ ^]^ to achieve simple rotational or bending motions. In addition, pneumatically activated systems usually lack autonomy, due to being tethered to a pump.^[^ ^14^ ^]^ Fully autonomous soft actuators exist, although these can suffer from fuel depletion upon long‐lasting activation.^[^ ^9^, ^15^ ^]^ These considerations limit the general use of pneumatic actuators. In contrast, systems driven by external fields generally perform better in terms of durable activity^[^ ^8^, ^16^ ^]^ and autonomy.^[^ ^17^, ^18^, ^19^ ^]^ This has led to field‐driven soft actuators, especially ones driven by magnetic fields, receiving significant attention recently,^[^ ^20^, ^21^, ^22^, ^23^, ^24^ ^]^ also in view of the range of forces that can be applied to transportable cargos using such systems.^[^ ^25^ ^]^

Fluid transport and mixing with whip‐like organelles, cilia, is an efficient soft actuation strategy found throughout nature: from microorganisms^[^ ^26^ ^]^ to mammalian airways^[^ ^1^, ^27^, ^28^ ^]^ to brain ventricles.^[^ ^29^ ^]^ In the microfluidic regime, viscosity dominates inertia, rendering conventional pumping of fluids highly inefficient. The efficiency of the ciliated fluid transport has motivated scientists to mimic this strategy, primarily to replicate the biological functionality.^[^ ^30^, ^31^, ^32^, ^33^, ^34^, ^35^ ^]^ Additionally, biomimetic cilia can be driven by external fields with relative ease.^[^ ^30^, ^31^, ^32^, ^36^, ^37^ ^]^ A recent demonstration thereof includes solid cargo transport employing electric fields in a liquid environment (silicone oil).^[^ ^38^ ^]^ The state of the art in transport using magnetic fields is the work on solid‐cargo transport using magnetic soft cilia.^[^ ^36^, ^39^ ^]^ However, these studies focused on micrometer‐sized colloidal objects suspended in a liquid. Only one example of solid cargo transport in a dry environment was reported thus far, namely for a viscoelastic cargo.^[^ ^40^ ^]^ Dry transport of other types of solids and, in particular, higher density solids, remains unexplored. Another recent study showed the transport and manipulation of water droplets using magnetically responsive board‐like structures.^[^ ^39^ ^]^ Yet, the fabrication of these boards is difficult to scale up and was limited to unidirectional transport. Many realizations of magnetically actuated artificial cilia are useful for specialized functionalities, but are often expensive or involve time‐intensive fabrication techniques, e.g., lithography,^[^ ^36^ ^]^ 3D printing,^[^ ^30^ ^]^ femtosecond laser writing,^[^ ^39^ ^]^ and manual assembly.^[^ ^32^ ^]^ This can significantly limit the scalability of these approaches.

Here, we create arrays of soft responsive pillars, which we collectively refer to as a soft magnetic carpet (SMC). Their manufacture is based on a hard ferromagnetic variant of the facile fabrication method outlined by Lu et al.^[^ ^41^ ^]^ and Timonen et al.,^[^ ^42^ ^]^ which involves an external magnetic field and a resin doped with magnetic particles (see the Experimental Section for details). Pillars emerge with the application of the field, due to the Rosensweig instability,^[^ ^43^ ^]^ and possess a permanent magnetic dipole moment after fabrication. A major advantage of this approach is its scalability. The permanent magnetic moment can be used to actuate the SMCs using a (patterned) external magnetic field. The use of silicone resins enables the “amphibious” transport of both liquids and solids, such as millimetric droplets and large objects, as well as the generation of fluid flows when the carpet is fully submerged. Dry transport can even take place for high‐density, heavy, and large objects. In addition, in both the dry and wet (submerged) states, there are nontrivial reversals of transport that we characterize theoretically and exploit for a number of applications including size‐ and shape‐dependent object separation. Owing to their simple design, scalable fabrication scheme, and the level of achievable fine control over transport, sorting, and mixing, we foresee a myriad of potential applications of these SMCs in industrial and academic settings.

## 2 Results

**Figure 1:**
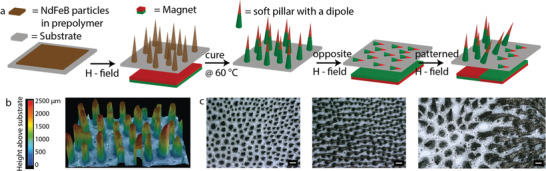
Fabrication of soft magnetic carpets. a) Sketches of the fabrication steps of our SMCs, depicting how the magnetic prepolymer forms pillar‐like structures that become stable after being cured at 60 °C. The pillars carry a permanent magnetic dipole moment and therefore respond to external magnetic fields. b) A reconstructed 3D reflection microscopy scan of the SMC; no magnetic field is applied here. c) Reflection microscopy images of the pillars sketched in (a), without an external magnetic field, with an oppositely directed field (compared to the formation), and with a half similarly and half oppositely directed field, respectively. Scale bars are 200 µm.

We fabricated an array of soft pillars with a permanent magnetization following a modification of the procedure outlined in,^[^ ^41^, ^42^ ^]^ see the Experimental Section for a full description. In brief, we spread a mixture of neodymium iron boron (NdFeB) particles (Magnequench MQFP‐B+, D50 ≈ 25 µm) and a soft silicone matrix (Ecoflex 00‐20) on a substrate (see **Figure** **1**a). To this layer, we applied a homogeneous magnetic field during the course of the polymerization. This caused the spontaneous formation of pillars due to a Rosensweig‐type instability;^[^ ^42^ ^]^ Figure 1b shows an image of the final shape. The size and density of the pillar array can be tuned readily by changing the applied field strength, the magnetic particle content, and the total amount of the magnetic mixture (see Table S1, Supporting Information, and its accompanying text for details of our fabrication and a comparison of our method to mold‐based techniques^[^ ^30^ ^]^). Once polymerized fully, the pillars respond to external magnetic fields, as they possess a permanent magnetic dipole moment imbued by the homogeneous external field during fabrication through the alignment of the moments of the individual NdFeB particles (Figure S1, Supporting Information, quantifies the degree of permanent magnetization). When an external field is applied parallel to the pillars’ dipole moment, the pillars stretch in the field gradient. When the magnetic field is oppositely directed, it bends the pillars onto the substrate surface (see Figure 1c). We found this response to be reversible, due to the pillars’ softness, and easy to tune on a millimetric scale by designing a magnetic actuation landscape, similar to the recent work.^[^ ^35^ ^]^ These features can be used to induce pillar motions that result in the transport of fluids and solids; here, through the application of a traveling oscillatory magnetic field. Such magnetic actuation allows one to perform work, as described next.

Our SMCs can be used to manipulate solid nonmagnetic objects in space. To demonstrate this, we arranged cylindrical magnets in a ring shape (magnetic track), alternating their orientation.

**Figure 2:**
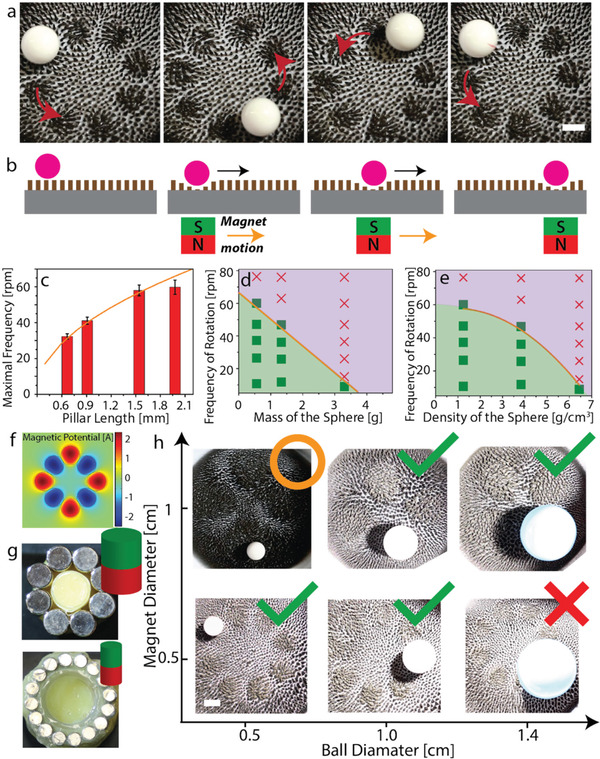
Micromanipulation of solid objects on dry soft magnetic carpets. a) Snapshots showing the circular motion of a spherical object by the rotation of a set of alternatingly oriented rod‐shaped magnets (magnetic track) placed underneath the substrate covered by the SMC. b) Sketches indicating the way the SMC behaves under the application of a localized magnetic field and how this induces transport of a sphere supported by the SMC. c) Pillar‐length dependence of the magnetic track's maximum angular frequency *ω* for which sphere transport occurs. d) State map of the sphere's ability to follow the motion of the magnetic track (green squares = 90% corotation, red crosses <50% corotation), plotted as a function of *ω* versus the sphere's mass. e) State map of *ω* versus the sphere's mass density. c–e) The orange curves show our theoretical prediction for the corotation crossover. f–h) Effect of the magnet size in relation to the sphere diameter on the transport performance. f) The gradient map provides the finite‐element calculated magnetic potential of the eight‐magnet track at the surface of the substrate showing that four natural wells will form. g) Photographs of the magnetic tracks. h) Photographs of the associated carpet patterns, with four and eight magnetic wells, respectively, and the spheres used. Green checks indicate corotation (less than 10% well hopping), the red crosses indicate more than 80% well hopping, and the orange circle indicates a roughly 50% rate of well hopping. All scale bars indicate 5 mm.

This magnetic track was rotated underneath the substrate covered by the magnetic pillars, on which a nonmagnetic millimetric sphere rested. The field induced by our magnetic track formed a ring‐shaped array of depressions (wells) on the surface of the carpet as shown in **Figure** **2**a,b. These wells were formed above places where the magnetic field was directed opposite to the orientation of the homogeneous field applied during fabrication, i.e., the intrinsic magnetization direction of the pillars. As such, there are half the number of depressions than there are magnets in the track (see Figure 2f,g). The wells were made mobile by rotating the track. They form a gravitationally favorable location for a solid object, as sketched in Figure 2b. Hence, the motion of the track causes the spheres to corotate, provided they do not escape the well (see Movie S1 in the Supporting Information). Here, the cargo remains in the same pocket during the transport. This implies that a minimum of three magnets should prove sufficient to achieve single‐cargo transport, but more magnets should be used for a higher level of control. Transport by this mechanism depends on several factors of which the most important are: the length of the pillars; the mass, density, and the size of the sphere; and the size and magnetic strength of the magnets. First, we analyzed the transport capacity of our SMCs as a function of the rotation frequency of the magnetic track, as well as pillar length. In all cases, we used a 9.5 mm sized sphere with a mass of 0.56 g. We plot the maximal angular frequency at which the sphere remains confined to its well in Figure 2c. The shorter the pillars (average height: 670 µm, 910 µm, 1.5 mm, and 2.0 mm, respectively), the less effective the wells were in retaining the sphere. Our SMCs feature pillars with a sub‐millimeter spacing, which implies that spatial manipulation of solid cargoes can take place on millimetric scales, noting that several pillars are required to define a well. Second, we used similarly sized spheres with different mass densities to explore their effect on the SMC's transportability (see the Experimental Section for details). We mapped successful (green points, less than 10% escapes) and unsuccessful rotations (red points, an escape rate exceeding 50%) in mass‐angular frequency (Figure 2d) and density‐angular frequency (Figure 2e) diagrams. Our SMCs were found to transport the lower mass and lower density spheres more readily. Finally, we have checked the effect of the ratio of magnet size to cargo size, i.e., the effect of the width of the well. For these experiments, we kept the pillar height constant at 2 mm. Figure 2h shows that matching the size of the magnet to the ball leads to better transport performance, compared to strong mismatches. A relatively small well cannot provide sufficient space to capture a large sphere. Conversely, when the well is much larger than the sphere, the sphere has significant freedom to move and can gain enough momentum in the well to jump over the edge.

We can understand the above result using a minimal model for the maximal work that can be harvested from our SMC system. In our model, we consider the maximum transport capacity to depend on three factors: i) pillar stiffness, ii) pillar susceptibility to the magnetic field, and iii) the pillar susceptibility to pressure. The former and latter two determine the height difference between the straight and bent pillars. We identify the potential energy difference between the sphere resting on the two pillar conformations as the main contributor to localizing an object (sphere) to a well (see Figure S2a in the Supporting Information). When the sphere's kinetic energy exceeds this potential energy gain, the sphere is expected to escape the well. Therefore, the magnetic track's maximum angular frequency *ω* at which corotation breaks down, is found by equating the sphere's kinetic (*K*) and potential energy (*P*), i.e., *K*  =  *P*. A sphere rotating with a constant frequency along the circular path imposed by the track has a kinetic energy $K=1/2\;mR^{2}\omega^{2}$, where *m* is the mass of the sphere and *R* is the track radius. The potential‐energy gain due to the magnetic deflection of the pillars is given by *P*_m_ =  *mg*Δ*h*_m_, where *g* is the acceleration due to gravity, Δ*h*_m_ is the height difference between the center of mass of the sphere resting on straight pillars and at the bottom of the well (see Figure S2b in the Supporting Information). A sphere is expected to make the deeper by its mass resting on the pillars. This gives rise to an additional potential energy difference *P*_w_ =  *mg*Δ*h*_w_, where Δ*h*_w_ is the mass‐induced height difference. We assume that the pillars act as a Hookian spring, *F*  =  *kx*, with *x* the deviation from equilibrium and spring constant *k* representing the stiffness of the silicon material (Ecoflex 00‐200) (see Figure S2c in the Supporting Information). Writing the magnetic force as *F*_m_, we obtain $\frac{1}{2}mR^{2}\;\omega^{2}=\frac{mg}{k}\;\left(F_{m}-mg\right)$. Solving this equation for *ω* results in the mass (and density dependences) shown by the orange curves in Figure 2c–e. In all cases, the shape agrees well with the experimental findings.

**Figure 3:**
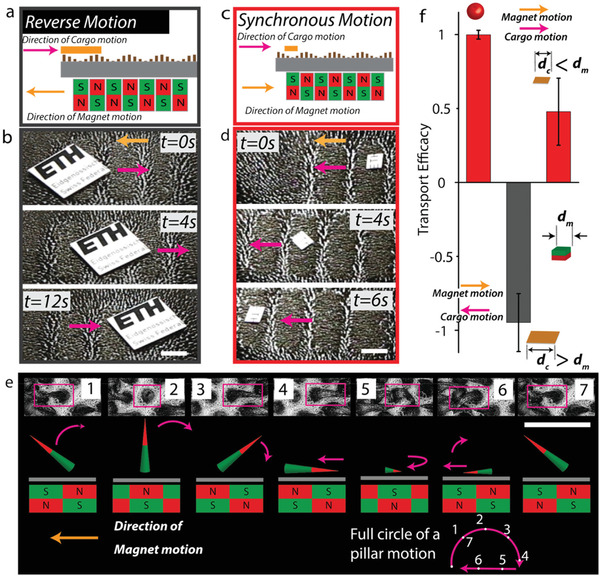
Size‐dependent transport on dry SMCs. a) Sketch of an SMC carrying the board‐shaped cargo in the opposite direction to the motion of the driving magnets. This demonstration is for an object with a contact‐dimension larger than that of a single magnet in the array. b) Time lapses showing the motion of a large board‐shaped cargo on an SMC. c) Sketch of an SMC carrying a small board‐shaped cargo along the same direction as the motion of the driving magnets. d) Time lapses showing the motion of a small board‐shaped cargo on an SMC. e) Time lapse of microscopy images showing a full cycle of pillar conformations during the motion of the magnet track toward the left. The top‐down images are accompanied by sketches indicating the position of the magnets in the track and the response of a single pillar. f) Transport efficacy (object motion relative to that of the magnetic track) for the spherical cargo and two sizes of board‐shaped cargos. Here *d*_m_ is the size of the magnet in a track and *d*_c_ is the size of the cargo. Scale bars indicate 5 mm in (b,d) and 500 µm in (e).

Our SMCs can transport a wide variety of shapes. The transport of a solid cargo depends sensitively on the shape of the cargo and in particular on the dimension of the object with respect to the natural wavelength of the wells in the SMC (see **Figure** **3**a–d). If the dimension of the contact is smaller than the magnetic‐well size, the cargo comoves with the magnets (see Figure 3c,d and Figure S3 and Movie S2 in the Supporting Information). However, intriguingly, for anisotropic shapes of sufficient size, like a cylinder or a board, cargo locomotion is opposite to that of the magnet track (see Figure 3a,b and also Movies S3 and S4, Supporting Information). To understand this behavior, we considered the motion of a single pillar as a function of the motion of the magnets, similar to what was done in the recent study.^[^ ^31^ ^]^ The time lapse in Figure 3e shows the associated sequence of pillar conformations. Here, microscopy images together with cross‐sectional sketches demonstrate that the pillar motion is highly anisotropic and covers a half circle (see inset Figure 3e). In a “forward stroke” (power stroke), steps 1–4, the pillar stretches and moves in the vertical direction, conversely in the “backward stroke” (return stroke), steps 5–7, the pillar shrinks and stays close to the substrate. We use these naming conventions to make the connection to the stroke pattern in biological cilia. Note that despite the naming, the forward stroke is, in fact, in the opposite direction to the motion of the magnet. Therefore, a cargo smaller than a single magnet unit (i.e., smaller than the magnetic well) travels in the same direction as the magnet. A cargo larger than a single magnet comprising the track does not fit inside the well and instead predominantly experiences the motion of the stretched pillars, which perform a power stroke, as depicted in steps 1–4. This causes such a large cargo to travel in the opposite direction to the magnetic track in a manner that is reminiscent of crowd surfing. The configurations shown in Figure 3e are representative of the dynamics of a single pillar. Other pillars subjected to this magnetic track are phase‐shifted in their dynamics by a factor that depends on their relative location to the center of the magnets. Transport efficacies for board‐shaped and spherical cargos are given in Figure 3f. It is clearly observed here that a board‐like cargo that is smaller than the magnets moves in the same direction as the magnets. However, a cargo that is larger moves in the opposite direction (there denoted as having a negative efficacy). The dual nature of the motion as a function of pillar contact can be used to separate out particles with different shapes and sizes. For instance, a ring‐shaped object can be sorted from spherical objects using our soft‐carpet setup (see Figure S4 and Movie S5 in the Supporting Information). Additional cargo shapes and associated transport efficiencies are also discussed in Figure S4 in the Supporting Information.

Solid cargo transport can also be performed over a tilted surface. We tilted the substrate at a 20° angle to the surface, in which case the soft carpet transported an alumina cylinder upward (as shown in Figure S3 and Movie S6 in the Supporting Information). The maximum slope over which transport may be achieved depends on the length of the pillars and its comparison to the size of the cargo. Generally, we expect that an increase of the ratio of pillar length to cargo size will result in improved performance on steeper slopes.

**Figure 4:**
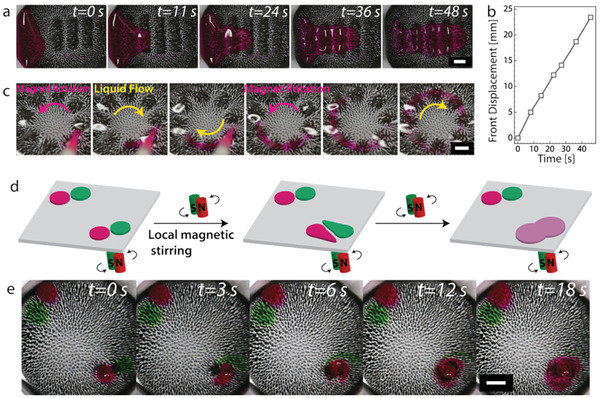
Droplet transport, fluid flow, and viscous mixing on SMCs. a) Photographic time lapse taken of a glycerol droplet (labeled with rhodamine) driven over a dry SMC by a linear track of magnets. The droplet moves in the opposite direction to the magnetic track. b) Plot of the displacement of the glycerol droplet's front with time. c) Continuous flow of glycerol (fully submerged SMC) driven by a circular magnetic track. The motion is in the direction opposite to that of the track. The direction of flow was visualized by adding a droplet of rhodamine labeled glycerol. d) Sketch demonstrating how soft carpets enable spatial control over local fluid mixing. Glycerol droplets labeled with different dyes (rhodamine and green food color) are placed at opposite corners of the millifluidic chip. Active mixing takes place in one corner while the fluid in the opposite corner remains effectively unperturbed. e) Photographic time lapse taken of a local mixing experiment that demonstrates the significant enhancement of SMC‐based mixing compared to that induced by diffusion in glycerol. Scale bars are 5 mm.

In addition to solid transport, our (otherwise dry) SMCs can transport and interact with liquid droplets. To demonstrate this, we placed a small amount of glycerol on one of our SMCs and labeled the fluid with rhodamine dye to visualize the transport. The time lapse shown in **Figure** **4**a demonstrates that the glycerol droplet moves in opposite direction to the motion of the magnetic‐field wave. Figure 4b quantifies the motion of the droplet front; the liquid moved over 23 mm in less than a minute, see Movie S7 in the Supporting Information. To induce a continuous flow, we implemented a ring‐shaped magnetic track and rotated this underneath an SMC fully submerged by glycerol. Figure 4c shows that this resulted in a liquid counter‐flow opposite to the direction of the track rotation, also see Movie S8 in the Supporting Information. This is reminiscent of antiplectic metachronal motion in biological cilia,^[^ ^31^, ^32^ ^]^ but as we will see shortly not necessarily indicative of metachronicity. Note that the direction of the movement, along or opposite to the track motion, depends on the liquid viscosity as explained in the subsequent sections.

Diffusive mixing of solutes in viscous liquids such as glycerol is generally inefficient due to the laminarity of the flow at low Reynolds numbers, i.e., viscous dissipation dominates inertia. This is a limiting factor in achieving conventional mixing in microfluidic chips, capillaries, and microfluidic devices.^[^ ^6^ ^]^ The nonreciprocal motion of biological and biomimetic cilia overcome this obstacle. Here, we demonstrate that our SMC are not only capable of achieving mixing at a microfluidic scale, but that this can be done with precise spatial control. We placed two droplets of glycerol with two distinct dye labels (rhodamine and green food color) in opposite corners of an SMC submerged in glycerol. A local stirring flow was induced at one corner using a pair of magnets. Over the course of 20–30 s, the droplets could be fully mixed by actuating the magnets, while the situation in the opposite corner remained relatively unperturbed (see Figure 4d,e and Movie S9 in the Supporting Information).

**Figure 5:**
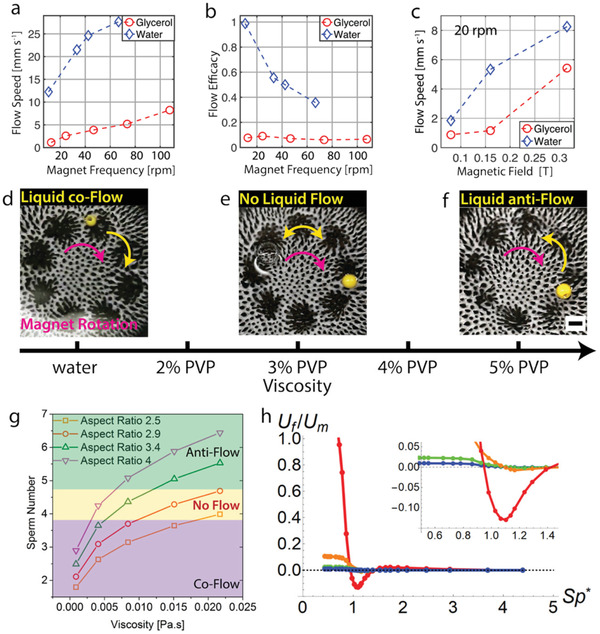
Nontrivial fluid transport properties. a) Dependence of the flow speed on the angular frequency of the magnetic track. b) Dependence of the transport efficacy on the angular frequency. c) Dependence of the flow speed to the magnetic field strength at affixed 20 rpm magnet frequency. d–f) Snapshots showing three instances of a sphere resting on a fluid layer that is actuated by a submerged SMC. The arrows show the direction of the magnetic track (purple) and the resultant liquid flow (yellow). The viscosity of the fluid increases from left to right leading to distinct flow regimes: d) DI water (comoving flow), e) 3% aqueous PVP solution (no net flow, stall‐flow), and f) 5% aqueous PVP solution (oppositely directed flow). g) Plot of the Sperm number, see main text for definition, for different aspect ratio magnetic pillars against the viscosity of the medium. h) Result of our bead‐spring model showing the time‐averaged fluid velocity of the interface *U*_f_ (reduced by the speed of an effective magnet on the circular track *U*_m_) as a function of the effective sperm number Sp* (where high Sp* corresponds to high viscosity and vice versa). From red to blue, the ratio of the cilium length *L* to fluid height *H* is *L*/*H* 2/3, 1/2, 1/3, 1/4, respectively. The inset shows the resulting flow reversal in detail. Scale bars are 5 mm.

Generally, wet SMCs induce a fluid flow whose speed depends on the magnetic track's angular frequency, the strength of the applied magnetic field, and the viscosity of the submerging fluid. The fluid flow was measured by tracking the motion of a sphere supported by the liquid–air interface and found to be proportional to the angular frequency (see **Figure** **5**a). The maximum flow speed generated was around 2.7 cm s^−1^ at an angular frequency of 60 rpm for water and it was about 0.8 mm s^−1^ for glycerol. The flow speed increased with increasing angular frequency, but appeared to plateau for our highest frequencies, this is reminiscent of some of the observations found in refs. [31, 35]. We also considered the flow efficacy. When the fluid makes one round for a single rotation of the magnetic track, the efficacy equals 1. Such efficiencies were only achieved at very low angular frequencies in water (around 10 rpm). The flow efficacy for glycerol was around 0.07 and remained relatively constant over the entire frequency range that we considered (see Figure 5b). Lastly, the flow speed increased by increasing the magnetic field applied to the wet SMC (see Figure 5c); we achieved this by adjusting the distance between the wet SMC and the magnetic track. We observed a flow reversal by switching from water to glycerol (see Figure 5d–f), analogous to the one reported in ref. [35]. We will explore this phenomenon next.

In a low‐viscosity medium, such as water, the flow was generally found to be in the same direction as the rotation of the magnetic track. This is reminiscent of symplectic ciliary transport in biology.^[^ ^44^, ^45^ ^]^ When using a sufficiently high viscosity liquid to submerge the SMC, such as glycerol, the flow was reproducibly found to be in the opposite direction. The possibility of such a reversal has been predicted for the nonreciprocal motion of a single (artificial) cilium and several (metachronally moving) cilia in various settings.^[^ ^46^, ^47^, ^48^ ^]^ Here, we go beyond the results of ref. [35] by examining a range of viscosities, we do so by preparing a series of aqueous polyvinylpyrrolidone (PVP, 360k) solutions up to 20% of PVP by weight (corresponding viscosities in Table S2, Supporting Information). For an SMC with a pillar aspect ratio of 3.1, the liquid flow in deionized (DI) water (0% PVP) was comoving (see Figure 5d and Movie S10, Supporting Information). This behavior persisted up to and including 2% PVP. For 3% PVP no net liquid flow was observed (stall flow) (see Figure 5e and Movie S11, Supporting Information). When the viscosity was further increased (5% PVP), net fluid flow was reestablished, but it was counter to track motion (see Figure 5f and Movie S12, Supporting Information). We further found that by changing the aspect ratio of the pillars of the SMC, the viscosity at which stall flow occurred could be shifted: increasing the aspect ratio decreases the viscosity at which the flow behavior changes. For very high aspect ratios, the flow was observed to be oppositely directed even in DI water. We quantify this counterintuitive behavior using the Sperm number *S*_p_. This is a dimensionless number characterizing the relative effect of viscous to elastic stresses on acting on a filament;^[^ ^49^ ^]^ see the Supporting Information for an estimate of this number. Figure 5g shows the effect of the aspect ratio on the flow behavior. Examining the state diagram of *S*_p_ as a function of viscosity, Figure 5g, reveals that the no‐flow region is found in narrow band around *S*_p_ ≈ 4 (see also Table S2 in the Supporting Information).

We will now provide arguments by which to understand this result. We first noted that the interface showed some undulation during the counter‐flow in the experiment. However, a dye test proved that the time‐averaged flow is indeed reversed (Figure 4c), indicating that the small oscillatory motion of the interface is not the mechanism by which the reverse transport of the bead occurs. That is, our energy‐based argument for transport over dry soft carpets cannot be applied to wet soft carpets. We instead understand the observed viscosity‐based transport reversal through a computational fluid dynamics study in the spirit of refs. [37, 47]. Details of the modeling are provided in Figures S5–S7 in the Supporting Information. More detailed analyses of cilia motion are possible, i.e., by adapting the work of,^[^ ^37^, ^47^ ^]^ however, this proved unnecessary for understanding the reversal. In brief, we use a hydrodynamic Greens’ function approach to capture the dynamics of the cilium, but we modify the hydrodynamic singularities to account for the finite height of the fluid layer following ref. [50]. This is necessary to show that any mobility reversal—already observed as a function of the sperm number close to a single cilium in a fluid half space^[^ ^37^ ^]^—still occurs at the surface of the confining fluid layer. The extension makes our model computationally more involved, and we, therefore, focus on the dynamics of a single sphere (bead) only, which is located at the tip of the cilium and represents its entire motion. This choice in modeling ciliary dynamics is inspired by ref. [51]. Our single bead is actuated to move along a closed path through an oscillatory driving that accounts for the presence of the magnets. We induced a nonlinear angular spring to prevent the bead from contacting the wall and a similar longitudinal spring to model the reorientation and extension/contraction of the cilium induced by the driving. This form of driving and choice of springs proved sufficient to capture the salient features of the experiment (see Figure 5h), which shows a reversal of the flow velocity with the sperm number for various heights of the fluid layer. The reversal can be understood by the interplay of a reduction in bead mobility near the surface, the restorative spring, and the interaction with the magnets. This is evidenced by the roughly unitary value of the effective sperm number Sp* at which the primary reversal occurs. We use a slightly different choice here than in the experiment^[^ ^52^, ^53^ ^]^ as is further clarified in the Supporting Information. At high viscosity, which corresponds to high Sp*, the cilium does not have a large deflection (as in the experiment), thus the net velocity of the interface comes purely from the asymmetry in mobility between the forward and backward strokes. The backstroke is favored here, because, while the mobility may be reduced closer to the wall, the amount of applied force is increased. At lower viscosities, i.e., lower Sp*, the cilium can maximally deflect, thereby coming close to the surface. This leads to a reweighting of the forward and backward components of the stroke. The reduced mobility close to the surface leads to there being a retention time, such that it takes a sufficiently long application of the magnet to push the bead away again. This retention eliminates the most powerful part of the return stroke, thus giving rise to net forward motion of the interface just above the anchoring point. Because we have only considered a single cilium here, the fluid velocity drops rapidly with a greater fluid height. This is unlike the experiment, wherein many cilia are simultaneously actuated across the surface. Nonetheless, our single‐cilium result reproduces the reversal and indicates that a metachronal pattern is not necessary for the generation of the mobility reversal in situations where the soft carpet is submerged, though we do not eliminate this possibility as a source of mobility reversal.

## 3 Conclusions

We have introduced soft magnetic carpets that consist of arrays of millimetric magnetic pillars that can be fabricated using a facile and scalable self‐assembly route. When actuated using a suitable external magnetic field, they can transport solid objects and liquid droplets as well as generate fluid flows for wireless microfluidic mixing. All of these can be controlled with a fine degree of spatial resolution, approximately millimetric here, using straightforward methods. Therefore, our system is readily advantageous to scientists with different levels of microrobotics experience, with potential for a wide range of applications in cell manipulation, biomechanical operations, or microfluidic pumping and mixing. Our finely structured soft matrix may be particularly beneficial for transporting fragile and delicate materials. Besides these key properties, we also found interesting forms of motion reversal both in the dry and wet state, which hold promise for additional applications and further degrees of control. In the dry state, transport reversal was shown to enable the sorting and separation of solid objects depending on their size and shape. Simple and fine control over the structure of the traveling magnetic field allowed our carpets to separate and sort a wide size and shape range of objects. In the wet state, a nontrivial flow inversion emerged by varying the viscosity. This has implications for achieving microfluidic viscosimetry and elastometry. We further showed that our carpets are able to transport objects up an incline. The combination of features demonstrated in our work lends soft magnetic carpets the potential for the transportation and sorting of fragile objects in industrial production and precision assembly lines.

## 4 Experimental Section

### Fabrication of the Soft Carpet

We modified the method of refs. [41, 42] to fabricate the soft, magnetic pillars. First, we prepared a mixture of Ecoflex 00‐20 (a Platinum Silicone rubber compound, Smooth‐on Inc.), hexane (Sigma‐Aldrich, ACS reagent), and magnetic neodymium iron boron particles (NdFeB, Magnequench MQFP‐B+, D50 = ≈25 µm, 10215‐088, Lot #F00492) with a weight ratio of 4:1:*x*, where *x* is set by the NdFeB particle concentration. In a typical experiment, we added 4 g of Ecoflex (2 g of each component), 1 g of hexane, and 3.6 g NdFeB powder (44 wt%). This mixture was homogenized in a Thinky mixer (ARE‐250) for 3 min at 2000 rpm. Next, 1 g of this mixture was poured into a Teflon petri dish (3 × 3 cm^2^, 0.25 mm thickness) and spread evenly by moving a permanent magnet (NdFeB, www.supermagnete.ch) back and forth underneath the petri dish, touching its bottom. Typically, the magnet had a dimension of 5 × 5 × 2 cm^3^ and a strength of 0.4 T. We could make different lengths and shapes of pillars by varying the strength of the magnet and the amount of mixture used. Once, pillars had formed, we increased the spacing between the magnet and the petri dish by 0.5 cm and moved this to an oven to be cured for 20 min at 60 °C, which solidified the Ecoflex 00‐20. A key requirement to obtaining a homogeneous SMC is to spread the mixture evenly before applying the magnetic field by which we induce the Rosensweig instability. By doing so, we were able to reproducibly obtain evenly sized pillars with a length polydispersity of less than 5%.

### The Magnetic Manipulation of SMCs

The magnetic manipulation of the SMCs was achieved by attaching a magnetic track directly to an electric motor or by utilizing a motorized stage to move the magnetic track. The pillar manipulation performed with the array of permanent magnets may in principle also be achieved using electromagnets that can generate similarly complex patterns of magnetic nodes and antinodes. However, to facilitate the use of electromagnets, it is recommended to magnetize the SMCs to their magnetic saturation level.^[^ ^30^ ^]^ This endows the pillars with a much greater permanent dipole moment, so that they can be more readily controlled using the typically weaker external fields generated electrically.

### Size and Density of the Spheres

The spherical balls used for dry SMC cargo transport experiments were as follows. For the experiments in Figure 2, 9.5, 8.72, and 9.96 mm balls with densities of 1.24 g cm^−3^ (polycarbonate), 3.83 (alumina) g cm^−3^, and 6.44 (zirconia) g cm^−3^ were used.

### Glycerol Labeling

To label the samples 30 mg of rhodamine isothiocyanate dye or 30 mg of green food coloring (Dr Oetker) was added to 1 mL glycerol. Small aliquots of this were dropped into the native glycerol to visualize the motion induced by the SMCs.

### Statistical Analysis

In Figure 2c, *Maximal frequency data* were determined by averaging five measurements and the data were displayed as mean ± standard deviation (M ± SD). In Figure 3f, *Transport efficacy data* were obtained via video analyses of four recordings for each data point and the data were displayed as M ± SD. In Figure 5a–c, Flow speed data were acquired by averaging three measurements and the data were displayed as mean values.

## Conflict of Interest

The authors declare no conflict of interest.

## Author Contributions

A.F.D. proposed and designed the research. D.Z. inspired the research. A.F.D., S.A., S.G., Y.M., E.P., J.A.C., C.Ü, and D.Z. performed the experiments and analyzed the data. R.H., J.d.G., and A.J.T.M.M. performed and analyzed the fluid dynamics simulations. A.F.D., J.d.G., and A.J.T.M.M. wrote the paper. All authors discussed the results and edited or commented on the paper.

## Supporting information

Supporting Information

Supplemental Movie 1

Supplemental Movie 2

Supplemental Movie 3

Supplemental Movie 4

Supplemental Movie 5

Supplemental Movie 6

Supplemental Movie 7

Supplemental Movie 8

Supplemental Movie 9

Supplemental Movie 10

Supplemental Movie 11

Supplemental Movie 12

## Data Availability

Data available upon request from authors.
