# Supplementary material for: Amphibious Transport of Fluids and Solids by Soft Magnetic Carpets
Source: Adv Sci (Weinh). 2021 Sep 16;8(21):2102510. doi: 10.1002/advs.202102510 (PMC8564456; doi:10.1002/advs.202102510)
Supplement: Supplementary file 1 — Supporting Information [file ADVS-8-2102510-s007.pdf]

© 2021 Wiley-VCH GmbH

ADVANCED SCIENCE
Open Access

Supporting Information

for *Adv. Sci.*, DOI: 10.1002/advs.202102510

"**Amphibious Transport of Fluids and Solids by Soft Magnetic Carpets** "

by Ahmet F. Demirörs[*,1], *Sümeyye Aykut*[1], *Sophia Ganzeboom*[1], *Yuki Meier*[1], *Robert Hardeman*[2], *Joost de Graaf*[2], *Arnold J. T. M. Mathijssen*[3], *Erik Poloni*[1], *Julia A. Carpenter*[1], *Caner Ünlü*[4], *and Daniel Zenhäusern*[5]
E-mail: ahmet.demiroers@mat.ethz.ch

Fabrication of soft magnetic pillars with various aspect ratios

Soft magnetic pillars with various aspect ratios were prepared for two sets of samples with different particle concentrations. For both sets the amount of Ecoflex was 4 g (2 g of each component) while the amount of NdFeB particles was changed from 3.6 g (47.4 wt%) to 6.6 g (62.3 wt%). All components were weighted and mixed in a cup and filled into a Teflon dish (area = 4.5x4.5 cm^2, thickness = 0.10 mm). To achieve different aspect ratios, the poured amount was varied from 0.5 to 2.5 g. The mixture was evenly spread by moving a permanent magnet (area = 5x5 cm^2, thickness = 2 cm, strength = 0.4 T, N pointing upwards, Supermagnete AG) and moving it back and forth and to the sides. Once the mixture was homogeneously distributed, the magnet was moved in circular motions to grow pillars. It is best to start from a distance of about 15 cm below the Teflon dish and to continue the circular motion while slowly moving upwards. After the pillars were formed, the magnet was placed underneath the sample with a spacing of 0.5 cm and cured in the oven at 60 °C for 20 min to solidify the Ecoflex.

Analysis of the length and aspect ratio of the pillars

The aspect ratio of the pillars was analyzed using an optical Keyence Microscope. With the 3D stitching software of the microscope, images containing about 40 pillars were recorded. 10 pillars were then chosen and their length and diameter were measured and averaged. These values are tabulated in Table S1.

Table S1. The length and aspect ratio of the pillars. The length of the magnetic pillars ranges from about 1000 μm up to 5 mm. The table provides the aspect ratio of the two sample sets with different magnetic-powder content. The aspect ratio decreases with increased thickness of the poured layer (exception: 0.5 g of 3.6 NdFeB).

3.6 g NdFeB	Amount filled in PTFE dish [g]	Height h [μm]	Radius r [μm]	Aspect ratio (h/2r)
	0.5	944.1	139.8	3.4
	1.5	2141.8	277.1	3.9
	2	3356.1	585.7	2.9
	2.5	3910.6	803.2	2.5
6.6 g NdFeB	0.5	3254.2	360.7	4.6
	1.5	3149.2	377.7	4.2
	2	5225.9	669.6	3.9

Comparison of fabrications methods: Pillars with Rosensweig instability vs molding

In this section, we contrast the fabrication method that we used with a common alternative method: molding. By utilizing the Rosensweig instability, we were only able to assemble conical pillars in a hexagonal fashion, not in a square fashion, for example. This is a disadvantage over a mold-based method, which allows for this type of arrangement[30]. However, our method does allow for control over the sample in a manner that circumvents the need for creating a new mold for each design, which may be a limiting factor in mold-based techniques. For instance, we may tune the strength of the applied magnetic field, the magnetic content of the elastic matrix, and the thickness of the mixture's spread before applying a field and subsequent curing. These parameters provide facile control over the height, spacing, and the homogeneity of the pillar lengths. Even spreading of the resin resulted in highly uniform pillar dimensions: <5% length polydispersity. Note that in relation to the pillars, we were able to obtain predominantly conical ones using our fabrication technique. Molding on the other hand allows for greater flexibility in terms of shape, such as the creation of cylinders[30]. Comparing these pillars, we expect that a conical shape will reduce the flow efficacy with respect to a cylindrical one, under the assumption that the pillars perform an otherwise identical cycle of front-and back- strokes. However, a thinner conical tip will likely bend further, thereby increasing the anisotropy between the strokes and potentially the (fluid) transport. It would be interesting to quantify this in the future. Another consideration in comparing both methods, is that molds can typically pillar a few centimeters squared surface, while our method allows for much larger areas to be reliably covered with

active cilia. Lastly, small pillar diameters will be difficult to demold, especially at high density, which is a limitation of that technique. Using the Rosensweig instability, we were able to reproducibly achieve pillar diameters of a few hundred microns at a relatively high density.

Permanent magnetization of the pillars

Note that the NdFeB particles are not magnetized to their magnetic saturation, instead they were used as received from the supplier. These NdFeB particles possessed an inherent magnetic anisotropy due to their shape or crystallinity, which caused them to assemble into chains along the external magnetic field applied during the formation of the pillars. The applied field was approximately uniform on the scale of the sample and directed orthogonal to the substrate on which the pillars were formed. After the matrix is cured this alignment is frozen in, which results in a permanent magnetic moment pointed along the long axis of the pillars. We show this in Supplementary Figure S1.

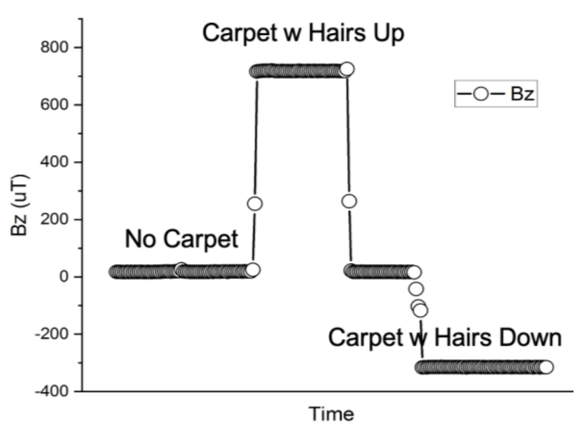

Figure S1. Carpet magnetization. Shown is the magnetic field strength measured using a magnetic sensor, when our SMC is placed 0.5 cm away from the sensor, in the absence of any externally applied fields. By flipping the soft magnetic carpet, the sign of the detected magnetic field is inverted.

Figure S1 shows the magnetic properties of our soft magnetic carpets. We plot the magnetic field detected normal to the direction of the SMCs surface (the direction of the pillars), using a magnetosensor (Adafruit LSM303AGR accelerometer magnetometer breakout). The SMC was placed 0.5 cm away from the sensor and flipped to show the presence of a magnetic moment. Figure S1 shows an associated sign flip of the magnetic field – the field is absent

when the carpet is not present. This indicates that the SMC has a permanent magnetic moment, which has a component in the direction orthogonal to the plane. We surmise that the pillars have the same magnetic properties as the whole and thus possess a magnetic moment along their long axis.

Model for Solid Cargo Transport

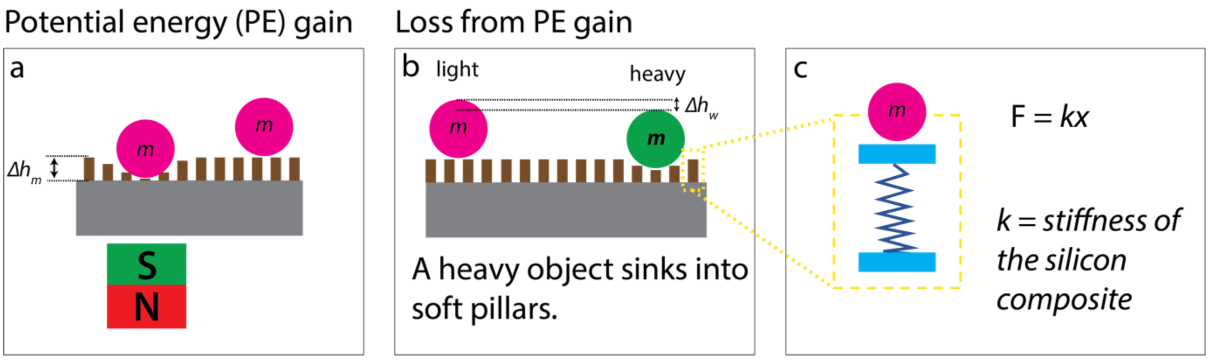

Figure S2. Minimal model for solid cargo transport on a SMC. a) The potential energy gain of a sphere sitting in a magnetic well can be estimated from the height difference Δh_m between the sphere inside the well and if it were resting on unperturbed pillars. b) A solid sphere will partially sink into the soft pillars, due to its mass, which improves the stability of the well. c) In our model we treat each pillar as a spring that obeys Hooke's law.

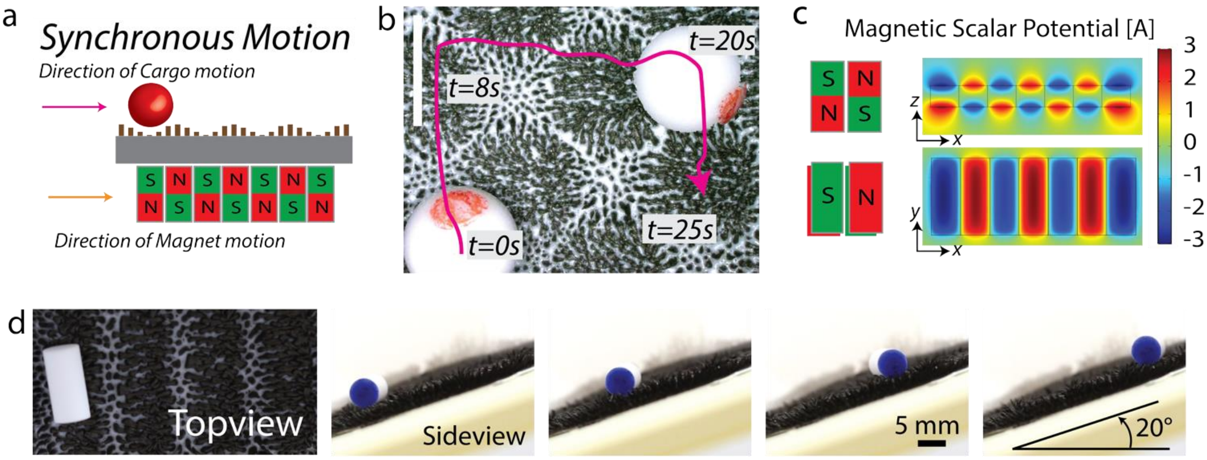

Figure S3. Solid Cargo Transport with SMCs over a Magnet set. a) Sketch of a SMC carrying a spherical cargo synchronized with the motion of the magnet. b) Microscopy

images taken at different time intervals showing the initial and final configuration of the sphere (the travelled path is indicated in pink), see also Supplementary Movie S3. c) Finite-element calculations and associated sketches of the magnet arrays used in (d) and Figure 4a (main text), showing the side view and top view of the magnetic potential. d) Transport of a cylinder over a ramp at an angle of 20° with respect to the horizontal. Scale bar indicates 5 mm.

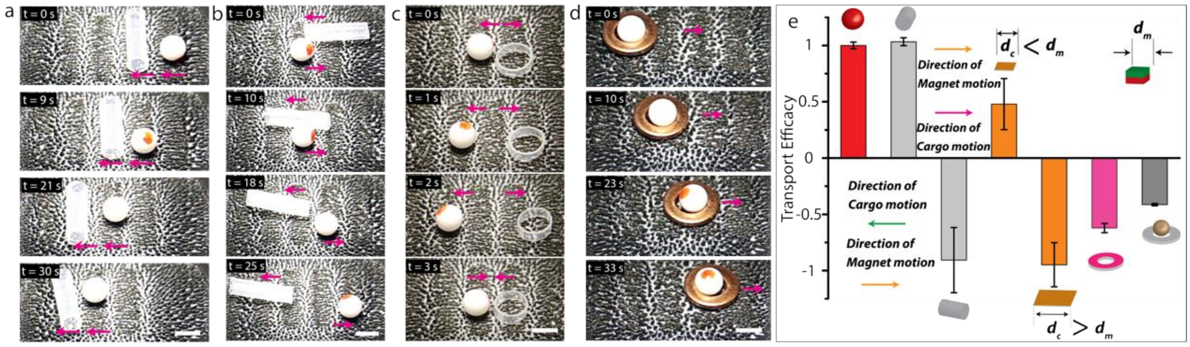

Figure S4. Solid cargo transport efficiency and particle separation. a) Two types of solid cargo, a rod and a sphere, are being transported in adjacent wells in the same direction. b) When the rod-shaped cargo is orthogonal to the magnetic wells it is transported in the direction opposite to the magnet and that of the sphere. This demonstrates a rudimentary particle separation mechanism. c) Oppositely directed transport of two types of cargos: a ring and a sphere. d) When a sphere is inserted inside a ring, a combined transport in the direction to that of the magnet occurs. That is, the crowd surfing ring dominates the motion. This coupling lowers the efficacy of the transport. e) An overview of all types of cargo shapes and their transport efficacy with respect to their synchronization with the magnetic field wave. Scale bars indicate 500 μm.

Here, transport efficacy is defined as the ratio between the motion of the object and that of the magnetic track resulting in a number between -1 and 1. When a cargo completely co-moves with the magnetic field wave, the efficacy is at its maximum value of 1. For a cargo that moves completely opposite to the motion of the magnetic field, the efficacy is -1. Different types of solid cargos were tested for their transport direction and efficacy, see Figure S4. The fact that the cargo particles move either same or opposite to the magnet

motion allows us a platform to separate particles by moving them in opposite directions. Examples of such separations are demonstrated in Figure S4b-c.

Sperm number estimation

We estimated the Sperm number using the expression $S_p = L/\left(\frac{\kappa}{\xi_\perp\ \omega}\right)^{1/4}$ where L is the length of the pillar, κ is its bending rigidity, ω the angular frequency of the magnetic field, and $\xi_\perp$ is the perpendicular drag coefficient. We consulted the paper of Dreyfus *et al.*[52] for these estimations. Sperm number depends on the viscosity of the liquid media and the aspect ratio of the pillars. The estimated Sperm numbers for various viscosities and various aspect ratio pillars are tabulated in Table S2.

Table S2. Viscosity and Sperm numbers. Viscosity of the liquid media and the estimated Sperm numbers for the various aspect ratio pillars in such media. Blue labeled viscosity highlights the stall-flow behavior.

	Aspect ratio →	2.4	2.9	3.4	4
	Viscosity	Sperm Number for NdFeB 3.6 g			
Water	8.90E-04	1.8	2.1	2.5	2.9
1% PVP	4.10E-03	2.6	3.1	3.6	4.2
2% PVP	8.40E-03	3.1	3.7	4.4	5.1
3% PVP	1.51E-02	3.6	4.3	5.1	5.9
4% PVP	2.17E-02	4	4.7	5.5	6.4
Glycerol	1.41E+00	11.3	13.3	15.7	18.3

The Computational Fluid Dynamics Study

Our model is comprised of a single sphere with unit diameter (we denote the radius with r) that is subject to four forces, which causes it to move along a trajectory that is representative of the cilium dynamics in the experimental system. The model is represented in Figure S5, which shows the three forces and torque acting on it. Here, $\boldsymbol{F}_d$ represents the periodic driving, hydrodynamic interaction with its surrounding leads to the hydrodynamic force $\boldsymbol{F}_h$, the elastic extension is captured by a non-linear spring force $\boldsymbol{F}_s$, and bending of the cilium is accounted for using a non-linear angular torque $\boldsymbol{T}$ that ensures that the bead cannot approach the surface too closely. We will explain our choices for the various forces next.

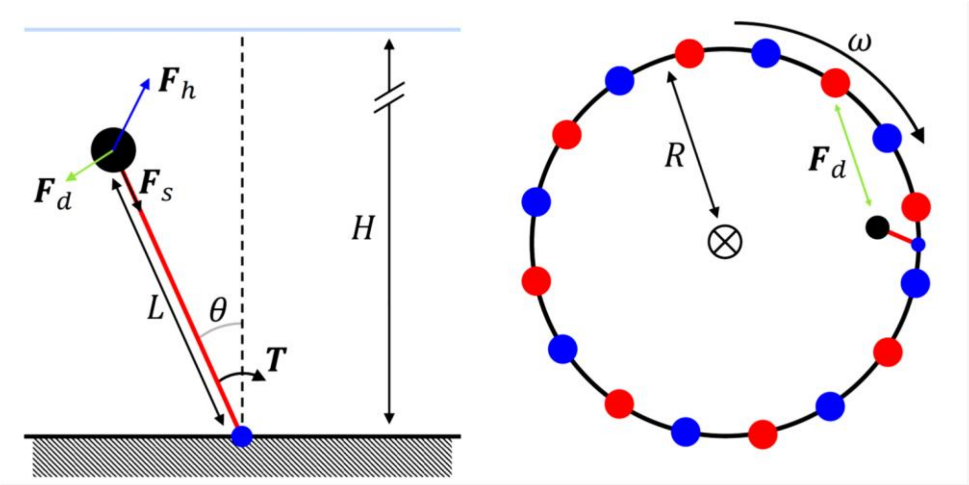

Figure S5: **Cartoon of the model cilium**. (left) Cartoon of the single-bead (black disk) model for the cilium, anchored (blue disk) to a no-slip wall (hashing) and bounded from above by a no-shear interface (light blue). A nonlinear angular torque $\boldsymbol{T}$ and spring force $\boldsymbol{F}_s$ (black arrows) cause the bead to move in a roughly semicircular path. The torque depends on the angle θ with respect to the z-axis. The bead is actuated using a periodic driving force $\boldsymbol{F}_d$ (green arrow) and it experiences a hydrodynamic drag due to the presence of a confined fluid medium $\boldsymbol{F}_h$ (blue arrow). (right) Cartoon representing the origin of the periodic driving force. Sixteen points, eight repulsive (blue) and eight attractive (red) push and pull on the bead, respectively, with cubically decaying interaction strength. These points move along a circular track with radius R and angular velocity ω leading to a net motion of the fluid layer directly above the cilium.

<u>The Hydrodynamic Boundary Conditions and Mobility</u>

We are interested in the dynamics of a cilium in a thin fluid layer. To model this, we consider a set of hydrodynamic boundary conditions representative of this situation. We place a no-slip surface at $z = 0$, i.e., a zero fluid velocity $\boldsymbol{u}(z = 0) = \boldsymbol{0}$, which represents the bottom surface of our setup. To account for the liquid-air interface, we impose a no-shear boundary condition at $z = H$. This requires that the derivative of the fluid velocity in the direction normal to the interface is zero at the interface. We determine the flow field and mobility of the particles in this system using a Greens' function approach that satisfies Stokes' equations, following Ref.[50]. There is no closed-form expression for the hydrodynamic monopole moment for this thin-fluid-layer geometry. We instead utilize a truncated series of hydrodynamic images using the standard Blake tensor image convention to approximatively account for the no-slip surface and a reflection to account for the no-shear surface. Here, we truncate the series to second order, such that the fluid velocity boundary condition at the interface is satisfied; a more significant truncation error is incurred at the bottom no-slip surface. The fluid velocity $\boldsymbol{u}$ is given by

$$\boldsymbol{u}(\boldsymbol{r}_f + \boldsymbol{r}_s) = \underline{\boldsymbol{g}}(\boldsymbol{r}_f, \boldsymbol{r}_s)\boldsymbol{F}_t \,,$$

where $\boldsymbol{r}_f$ is a point in the fluid, $\boldsymbol{r}_s$ is the point where the force is applied, $\underline{\boldsymbol{g}}(\boldsymbol{r}_f, \boldsymbol{r}_s)$ is the approximate Greens' function, and $\boldsymbol{F}_t$ is the total force acting on the point $\boldsymbol{r}_s$. Figure S6 shows the resulting flow field due to a mid-channel point force oriented along and perpendicular to the surface, respectively. Note that the streamlines follow the top surface, as required by the no-shear boundary condition.

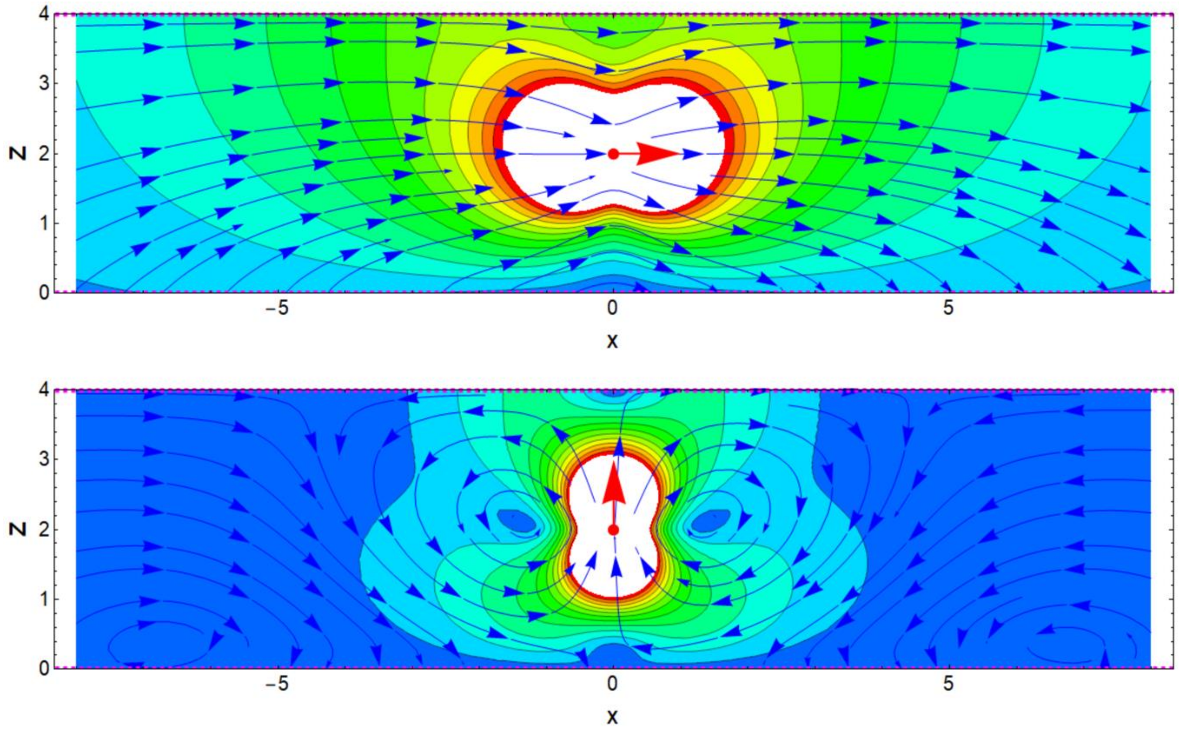

Figure S6: **Flow fields generated by the cilium**. Two-dimensional *xz*-slice through the three-dimensional flow field generated by a point force confined between a solid wall with no-slip boundary condition ($z = 0$) and a no-shear fluid-gas interface ($z = 4$); these are indicated using magenta dashed lines. The flow fields have been computed up to the second order to ensure that the boundary condition at the liquid-air interface is well respected. The top panel shows the flow field around a mid-plane force directed along the *x*-axis (red arrow), whilst the bottom panel shows the same situation with the force directed along the *z*-axis. The blue arrows indicate streamlines, while the color gradient indicates the fluid velocity magnitude, which is increasing from blue to red. In the white region, the flow speed exceeds our cut-off for visualization.

We derived the self-interaction and pair-interaction Rotne-Prager-Yamagawa-type mobility tensors from our monopole flow field Greens' function using the method outlined in Ref. [37]. The process of converting this into a functional and efficient hydrodynamic code is explained in the files and notes enclosed in our accompanying open-access data package. The Mathematica notebook therein may be used to derive higher-order corrections to the Greens' function. However, the compilation and calculation time do not scale favorably with increasing numbers of reflections.

The dynamics of a single bead may be written as

$$\dot{\boldsymbol{r}}(t) = \underline{\boldsymbol{\mu}}(\boldsymbol{r}(t))\boldsymbol{F}_t(t)\,,$$

where $\dot{\boldsymbol{r}}(t)$ is the time derivative of the beads position vector (as a function of time t), $\underline{\boldsymbol{\mu}}(\boldsymbol{r}(t))$ is the self-mobility tensor that depends on the position of the bead within the channel, and which coverts the total force $\boldsymbol{F}_t$ into a velocity. It should be noted that the source code provided with this paper provides additional mobility tensors for pair interactions between beads.

Linear and Angular Spring:

We capture the stretchability and bending of the cilium for our single-bead model using a linear and angular spring, respectively. We ensure that the deviation of the tip is not too significant (the stretching of the cilium is limited) and the tip does not scrape the no-slip surface by imposing finite-extensible non-linear elastic (FENE) responses. We should note that we also explored a version of the multi-bead approach of Refs. [37,47], to be reported on in future work. However, this model proved to be unstable for the significant bending observed in experiment, due to the choice of a dot-product-based angular potential between the beads. In the experimental system, the tip of the cilium scrapes the bottom surface, which is a hydrodynamically challenging situation to resolve and which can strongly limit the numerical stability of any algorithm. These considerations prompted us to utilize the following FENE form for the torque:

$$\boldsymbol{T} = K_a \frac{\theta}{1 - (\theta/\theta_0)^2}\hat{\boldsymbol{e}}$$

in our modeling. Here, θ is the angle between the cilium and the normal vector to the plane, $\theta_0 = 1.25 \approx 0.4\pi$ is the maximum permitted deviation (sufficiently small to keep the single bead away from the wall), K_a the bending prefactor, and $\hat{\boldsymbol{e}}$ the unit vector that can be constructed from the cross product of the orientation of the cilium ($\hat{\boldsymbol{l}}$ being the unit vector pointing from the anchoring point to the position of the cilium-representing bead) and the normal of the plane $\hat{\boldsymbol{z}}$, i.e., $\hat{\boldsymbol{e}} = \hat{\boldsymbol{l}} \times \hat{\boldsymbol{z}}$. We convert the torque into a force acting on the bead using the lever principle. That is, the torque can be represented as a force $\boldsymbol{F}_a$ acting on the tip of an arm equal in length to the distance between the anchoring point and the cilium bead. The direction is then determined using the cross product of $\hat{\boldsymbol{e}}$ and $\hat{\boldsymbol{l}}$.

The expression for the linear FENE force on the bead, which keeps it from moving too far away from the anchoring point, is given by

$$\boldsymbol{F}_s = K_l \frac{\Delta L}{1 - (\Delta L/\Delta L_0)^2} \hat{\boldsymbol{l}},$$

where K_l is the spring constant, ΔL is the deviation from the spring's equilibrium length, and ΔL_0 is the maximum permitted deviation from the equilibrium length. We chose $K_l = K_a$ and ΔL_0 to be 5% of ΔL_0 in our calculations. This ensures that our bead followed a closed, area-enclosing path through phase space, necessary for the generation of a net fluid flow[53]. Typically, our model cilium is elongated during the forward stroke, whilst being compressed during the back stroke. The effectiveness of the stroke can be increased by increasing ΔL_0. The speed of the two parts of the stroke is different, as we will also see shortly.

The Periodic Forcing:

To model the force of the magnets acting on the cilium, we account for a periodic driving. Here, we apply the form

$$\boldsymbol{F}_d = K_m \sum_{i=1}^{16} \operatorname{sign}(i) \left(\frac{2r}{|\boldsymbol{p}_i|}\right)^3 \hat{\boldsymbol{p}}_i,$$

where K_m is the magnetic strength, and $\boldsymbol{p}_i$ the position difference between the cilium bead and the i-th out of 16-point forces that model the 8 magnets:

$$\boldsymbol{p}_i = \boldsymbol{r}(t) - \begin{pmatrix} R\sin(2\pi i/16 + \omega t) \\ R\cos(2\pi i/16 + \omega t) \\ 0 \end{pmatrix}$$

for which ω represents angular velocity of the magnetic track and t is the time. We model the decay of the magnetic strength using an inverse cubic scaling here, though other choices can be made. It should be noted that this model does not accurately capture the exact nature of the magnetic field or the motion of the cilium carpet. However, it shows that the features observed in the experiment are robust to significant changes, which is satisfying from the perspective of implementing variants of our soft magnetic carpets. That is, the motion reversal will presumably persist.

Results:

Together, the above considerations give rise to the following expression for the net force acting on a single bead $\boldsymbol{F}_t = \boldsymbol{F}_a + \boldsymbol{F}_s + \boldsymbol{F}_d$, for which the steady-state dynamics is shown in Figure S6. This figure visualizes the motion of the cilium in the low-viscosity co-flowing

state and the high-viscosity counter-flowing state. We converted the viscosity η in our calculations to an effective Sperm number

$$Sp^* = L\left(\frac{(6\pi\eta a)\omega}{K_a}\right)^{1/4},$$

where L is the equilibrium length, a is the radius of the bead and $\omega = 2\pi/T$. This is a slightly different expression from that used in describing the experiment in the main text. However, this is justified as it only relies on parameters that are present in the simulation; we do not have a rod-like mobility, as is the case in the experiment.

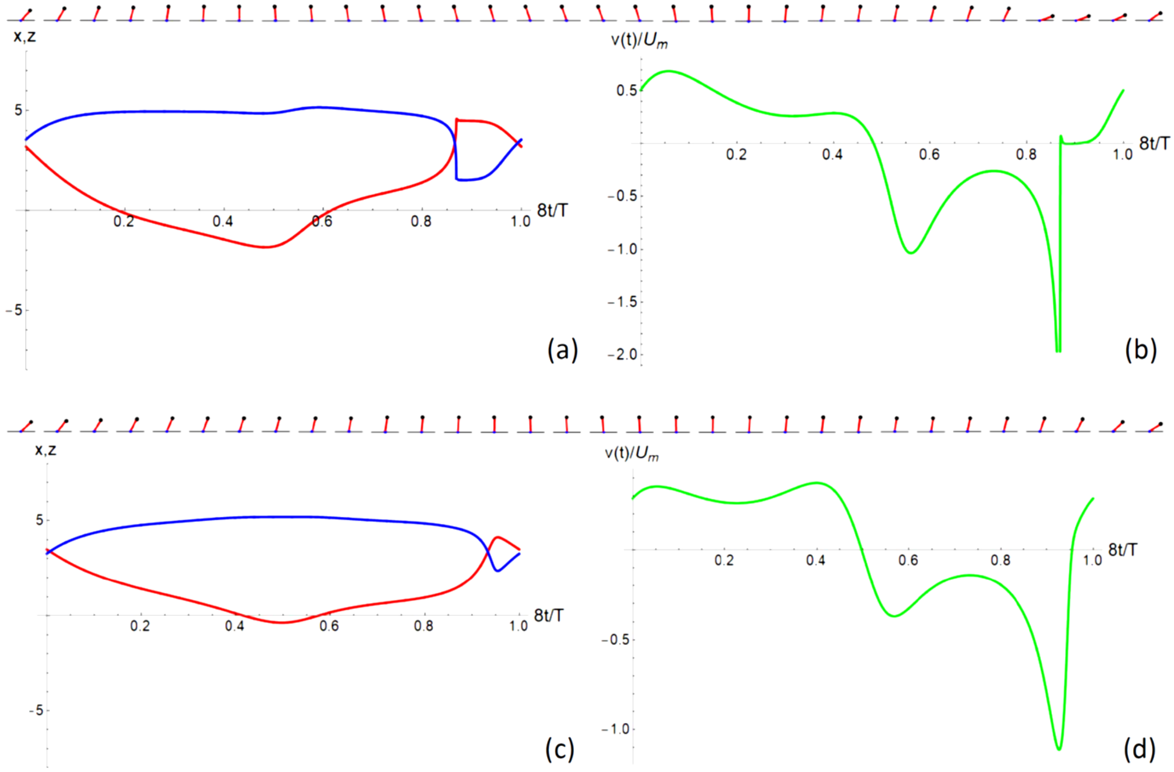

Figure S7: **Dynamics of the cilium at different Sperm numbers**. Visualization of the dynamics of our bead-spring cilium model for a fluid layer of height $H = 2L$, with L the cilium length and two sperm numbers $Sp^* = 1.0$ (a,b) and $Sp^* = 1.2$ (c,d), corresponding to the situations of forward (low viscosity regime) and backward flow (high viscosity regime), respectively. Note that positive $v(t)$ corresponds to motion in the direction of the model magnets. Subfigures (a,c) show the position of the bead in the xz-plane as a function of the time it takes one of the eight 'magnets' (an attractor-repulsor pair) to pass underneath the cilium. The red curve shows the x-coordinate, whilst the blue one shows the z-coordinate. Subfigures (b,d) show the instantaneous velocity $v(t)$ of the fluid interface directly above

where the cilium is anchored as a function of the same reduced time as in (a,c). The velocity has been reduced by that of the magnets U_m. The two strips above the sets of panels show 32 snapshots of the cilium taken equidistantly over the period of motion; forward motion is toward the right. The thin black line indicates the surface, the blue dot the position of the bead, and the red line connects the bead to its anchoring point.

From Figure S7 it becomes clear that within our simple model, the transition from co-fluid motion to counter-fluid motion is due the interplay between the driving and elastic properties of the cilium. The reversed motion relies on the cilium achieving its maximum angular extent for some time, as the model magnetic track runs underneath it. The rapid motion toward the surface is indicative of the attractive part of the 'magnetic' field moving in unison with the bead. This causes the bead to be pushed close (or the tip of the pillar in the experiment even onto) the surface. The reduced mobility close to the surface leads to there being a retention time, as only the repulsive 'magnetic' interaction is sufficient to push the bead away. This retention eliminates the most powerful part of the return stroke, thus giving rise to net forward motion of the interface just above the anchoring point. In the case of a greater viscosity, the motion toward the surface cannot be made fully, leading to less significant effects of near-wall mobility reduction. That is, the period of slow-down that is visible in Figure S7b is nearly absent in Figure S7d. Here, the forward motion loses out against the backward motion, as the return stroke may be entered in a lower-mobility regime (closer to the wall), but it the force closer to the wall is more substantial. The secondary (weak) motion reversal, not shown here, is caused by the linear extensibility of the cilia dominating the physics of our model at even higher viscosities, *i.e.*, when the deflection is minimal. We believe this to be predominantly a result of our choices of modeling the magnetic track, which we will explore in future work. Lastly, it should be noted that it is the net fluid motion of the interface above the cilium that reverses, rather than the motion between cilia, as can occur when multiple cilia are modelled[28].

Supplementary Movies

Movie S1. Circular transport of a sphere on dry soft magnetic carpet.
Movie S2. Transport of a sphere on a rectangular path on dry soft magnetic carpet.
Movie S3. Cycle of single cilium motion with the travelling magnetic field.
Movie S4. Linear transport of a board-shaped cargo on dry soft magnetic carpet.
Movie S5. Particle separation on dry soft magnetic carpet.
Movie S6. Uphill linear transport of a cylinder on dry soft magnetic carpet.
Movie S7. Linear Transport of a glycerol droplet on soft magnetic carpet.
Movie S8. Flow generated by the wet soft magnetic carpet in glycerol.
Movie S9. Spatially controlled liquid mixing with wet soft magnetic carpet in viscous medium.
Movie S10. Comoving liquid flow with wet soft magnetic carpet in DI water.
Movie S11. Stall-flow of the liquid on the wet soft magnetic carpet in an intermediate viscosity medium.
Movie S12. Oppositely-directed liquid flow generated with the wet soft magnetic carpet in a viscous medium.
